# A genome-wide linkage study of mammographic density, a risk factor for breast cancer

**DOI:** 10.1186/bcr3078

**Published:** 2011-12-21

**Authors:** Celia MT Greenwood, Andrew D Paterson, Linda Linton, Irene L Andrulis, Carmel Apicella, Apostolos Dimitromanolakis, Valentina Kriukov, Lisa J Martin, Ayesha Salleh, Elena Samiltchuk, Rashmi V Parekh, Melissa C Southey, Esther M John, John L Hopper, Norman F Boyd, Johanna M Rommens

**Affiliations:** 1 Department of Oncology (Division of Cancer Epidemiology), and Department of Epidemiology, Biostatistics and Occupational Health, McGill University, Montreal, QC; Lady Davis Research Institute, Centre for Clinical Epidemiology and Community Studies, Jewish General Hospital, 3755 Côte Ste-Catherine, Montreal, QC H3T 1E2 Canada; 2 Program in Genetics & Genome Biology, The Hospital for Sick Children, 101 College Street, East Tower, Toronto, ON M5G 1L7 Canada; 3 Dalla Lana School of Public Health, University of Toronto, Toronto, ON M5T 3M7 Canada; 4 The Campbell Family Cancer Research Institute, Toronto, ON M5G 2M9 Canada; 5 Ontario Genetics Network, Ontario Cancer Care, Toronto; Samuel Lunenfeld Research Institute and Department of Pathology & Laboratory Medicine, Mount Sinai Hospital, Toronto, ON M5G 1X5 Canada; 6 Department of Molecular Genetics, University of Toronto, Toronto, ON M5G 1A8 Canada; 7 Center for Molecular, Environmental, Genetic and Analytical Epidemiology, School of Public Health, The University of Melbourne, Melbourne, Melbourne, Victoria 3053, Australia; 8 Department of Medical Biophysics, University of Toronto, Toronto, ON M5G 2M9 Canada; 9 Department of Pathology, The University of Melbourne, Melbourne, Melbourne, Victoria 3053, Australia; 10 Department of Health Research and Policy, Stanford University School of Medicine and Stanford Cancer Center, Stanford; Cancer Prevention Institute of California, Fremont, CA 94538, USA

## Abstract

**Introduction:**

Mammographic breast density is a highly heritable (h^2 ^> 0.6) and strong risk factor for breast cancer. We conducted a genome-wide linkage study to identify loci influencing mammographic breast density (MD).

**Methods:**

Epidemiological data were assembled on 1,415 families from the Australia, Northern California and Ontario sites of the Breast Cancer Family Registry, and additional families recruited in Australia and Ontario. Families consisted of sister pairs with age-matched mammograms and data on factors known to influence MD. Single nucleotide polymorphism (SNP) genotyping was performed on 3,952 individuals using the Illumina Infinium 6K linkage panel.

**Results:**

Using a variance components method, genome-wide linkage analysis was performed using quantitative traits obtained by adjusting MD measurements for known covariates. Our primary trait was formed by fitting a linear model to the square root of the percentage of the breast area that was dense (PMD), adjusting for age at mammogram, number of live births, menopausal status, weight, height, weight squared, and menopausal hormone therapy. The maximum logarithm of odds (LOD) score from the genome-wide scan was on chromosome 7p14.1-p13 (LOD = 2.69; 63.5 cM) for covariate-adjusted PMD, with a 1-LOD interval spanning 8.6 cM. A similar signal was seen for the covariate adjusted area of the breast that was dense (DA) phenotype. Simulations showed that the complete sample had adequate power to detect LOD scores of 3 or 3.5 for a locus accounting for 20% of phenotypic variance. A modest peak initially seen on chromosome 7q32.3-q34 increased in strength when only the 513 families with at least two sisters below 50 years of age were included in the analysis (LOD 3.2; 140.7 cM, 1-LOD interval spanning 9.6 cM). In a subgroup analysis, we also found a LOD score of 3.3 for DA phenotype on chromosome 12.11.22-q13.11 (60.8 cM, 1-LOD interval spanning 9.3 cM), overlapping a region identified in a previous study.

**Conclusions:**

The suggestive peaks and the larger linkage signal seen in the subset of pedigrees with younger participants highlight regions of interest for further study to identify genes that determine MD, with the goal of understanding mammographic density and its involvement in susceptibility to breast cancer.

## Introduction

Mammographic density (MD), adjusted for age and body mass index (BMI) is a strong risk factor for breast cancer. The radiographic appearance of the breast on mammography varies among women and reflects differences in breast tissue composition and X-ray attenuation characteristics [1]. Fat is radiologically lucent [1] and appears dark on a mammogram, whereas connective and epithelial tissues are radiologically dense and appear light. The area of radiological dense tissue is often expressed as a percentage of the total breast area (percent mammographic density (PMD)). Compared to women of the same age and BMI with little or no density, PMD of ≥75% is associated with a four- to five-fold increased risk of breast cancer and an increased risk of all of the proliferative lesions that are thought to be precursors of breast cancer [2]. The increased breast cancer risk associated with high PMD does not differ by age, menopausal status, or race/ethnicity and cannot be explained by the 'masking' of cancers by dense tissue [3]. Extensive MD is relatively common and estimates of the associated attributable risk suggest that about a third of breast cancer may be explained by density in more than 50% of the breast [4,5].

Twin studies have shown that, after adjustment for other factors such as age, parity, menopausal status, body weight and hormonal use, about 60% of the variance in PMD is explained by genetic factors [6,7]. Both the dense area of the mammogram and the non-dense area have been found to be heritable to a degree similar to PMD. About two thirds of the negative correlation between dense and non-dense area was explained by the same genetic factors influencing both traits, but in opposing directions [8]. To find genetic determinants of variation in MD, we have carried out a genome-wide linkage study with over 1,400 nuclear families with the aim of examining previously reported loci and of identifying new loci that influence MD.

## Materials and methods

### Recruitment of study subjects

Families consisting of at least two sisters were assembled from several family registries and cohorts: families from Ontario, Australia and Northern California enrolled in the Breast Cancer Family Registry (BCFR) [9], dizygous (DZ) twins participating in twin studies in Ontario and Australia [6], families recruited in Ontario for a study of MD in young women, families from Ontario recruited through the 'Weekend to End Breast Cancer' event, and twins recruited through the Ontario Breast Screening Program (Table 1 and Additional file 1: supplementary table 1). Families from the BCFR known to carry mutations in BRCA1or BRCA2 were excluded; other participants were not screened. Epidemiologic data on relevant covariates, previously obtained by questionnaire within each of the above mentioned studies, were extracted from existing databases and assembled. Approval for the study was given by the Research Ethics Board of The Hospital for Sick Children, the Institutional Review Boards of the Cancer Prevention Institute of California and the University Health Network, Toronto, and The Human Research Ethics Committee of the University of Melbourne. Informed written consent was obtained from each participant. Participant recruitment from base populations is described and summarized in Additional file 1: supplementary Figure 1. In Ontario 1,137 (27%) of 4,126 subjects contacted had complete data and were included in the analysis. In Northern California 579 (38%) of 1,511 subjects contacted were included in the analysis. A total of 1,537 subjects were included from Australia from recent and ongoing recruitments that included collection of mammograms.

**Table 1:** Recruitment of families for linkage

Study name	Families, n (%)	Women with complete data for analysis, n (%)
All Families	1,415 (100)	3,253 (100)
Australian site of the BCFR	70 (5.0)	174 (5.4)
Australian Twin & Sister Study	589 (41.6)	1,363 (41.9)
Northern California site of the BCFR	257 (18.2)	579 (17.8)
Ontario site of the BCFR	246 (17.4)	561 (17.3)
Ontario families from other sources	253 (17.9)	576 (17.7)

BCFR, Breast Cancer Family Registry.

### Measurement of mammographic density

Mammograms were obtained from at least two sisters in each family. If a woman had breast cancer, the selected mammogram (from the contra breast) had been taken at or before the diagnosis of breast cancer. We sought that the ages at mammogram for two sisters should be within 5 years of each other to minimize the requirement for age-adjustment within a sibship. This was achieved in more than 95% of the families. The cranio-caudal view in each mammogram was digitized and sent to Toronto where all images were measured by one reader (NFB) using a computer-assisted thresholding method. Using Cumulus software, thresholds were set that defined the edge of the breast and outlined the areas of dense tissue. The pixels within these thresholds defined, respectively, the total area of the breast and the area of dense tissue (DA), from which percent density (PMD) was calculated. The non-dense area (NDA) of the mammogram was also calculated from these measurements [10]. This measurement method has been used to define differences in risk of breast cancer associated with mammographic density [2-5] and generated the evidence of heritability that motivated the present study [6].

Images were measured in batches of approximately 100 images at a time. Within each batch, 10 images were duplicated, placed in random order within the set, and read twice in a blind fashion. Some images were also re-read in different batches, in order to assess reliability of the PMD measurements both within and between batches. The correlation between the two reads in the same batch for PMD was estimated to be 0.90 (Additional file 1: supplementary Figure 2) and correlation for DA was 0.92. In addition, there were 10 images that were scored three times in three different batches, and the intraclass correlation between batches was estimated to be 0.902 by variance components analysis.

### DNA, genotyping and data cleaning

DNA was obtained from the BCFR biorepositories or was extracted from whole blood or lymphoblastoid cell lines using Gentra Puregene Blood Kit (Qiagen, Inc., Toronto, ON, Canada) or QIAamp DNA Blood Maxi Kit (Qiagen) according to the supplier's instructions. DNAs were quantified with Quant-iT PicoGreen dsDNA Reagent (Life Technologies, Inc., Burlington, ON, Canada) using fluorimetry measured with a SpectraMax Gemini EM instrument (Molecular Devices).

Genotyping for linkage analysis was performed by the Center for Inherited Disease Research (CIDR) http://www.cidr.jhmi.edu using the Illumina Infinium II Human Linkage-12 panel. Out of 6,090 SNPs, 413 were removed due to poor clustering, leaving 5,677 SNPs with an overall genotype missing rate of 0.073%. After examining Mendelian errors and estimating relationships [11-13], 49 pedigrees were modified to create half siblings when necessary, 6 larger pedigrees were created by merging two families (this information was subsequently confirmed from source site), 1 family was excluded due to sisters appearing unrelated, two families were removed due to a sample mix-up, and one sibling that appeared unrelated was removed from a larger pedigree (Additional file 1: supplementary Figure 3). After adjusting the pedigree structures, genotypes demonstrating Mendelian inheritance inconsistencies [14] were set to missing for all individuals in the families concerned; this involved removing a total of 475 genotypes on the autosomes. We removed all X chromosome data for six women where all genotypes were homozygous or had unusually high rates of missing genotypes.

Genotypes were missing in less than 20 individuals for 97.4% of the markers; and the poorest marker failed in 81 of 3,952 individuals. Ten or fewer marker genotypes were missing in 91% of the genotyped individuals. Allele frequencies were estimated from all genotyped individuals. Only 1.9% of the markers had minor allele frequency (MAF) less than 0.10, and 76% of the markers had MAF over 0.3; average heterozygosity across SNPs was 43.7%. Multipoint marker informativity remained very stable across the genome, ranging from 0.6 to 0.8.

Although self-reported race/ethnicity was available, we estimated population structure by using Eigenstrat [15] on one individual per family, combined with 1,207 HapMap phase 3 samples [16] from 10 populations, and our 5,677 SNPs. SNP alleles were flipped when necessary to match the strand used by CIDR. We found, as expected, that the majority of the samples overlapped with the HapMap CEU group, and furthermore that self-reported race/ethnicity matched well with clusters of Asian and African descent from the HapMap samples (Additional file 1: supplementary figure 4). Principal components (PCs) were estimated for all study participants from the eigenvectors, and then a Caucasian subgroup was defined as those with the first PC ≥0.003 and the second PC ≥0.

Physical marker locations were obtained from NCBI Build 36 http://www.ncbi.nlm.nih.gov/. The Rutgers linkage map was obtained from Rutgers Map Version 2 http://compgen.rutgers.edu/[17]. For markers not included in the Rutgers data set, genetic distances were interpolated at the Rutgers site.

Genotyping of individual single-nucleotide polymorphisms (SNPs) to attempt validation of association was performed with allele-specific fluorescent probes in Taqman^® ^SNP Genotyping Assays (C_334499_10 for rs723149 from Applied Biosystems, Foster City, CA, USA) as recommended using a 96-well format. End-point fluorescence was measured with the plate reader component of the 7900HT Real Time PCR System (Applied Biosystems) and aided by Taqman^® ^Genotyper software for allele discrimination with call rates > 98%. A portion of the samples (4%) was run in duplicate and corresponded to individuals used in the linkage study to assure quality control and permit assessment across genotyping platforms. The concordances of replicate genotypes and cross platform genotypes were > 99%.

### Statistical analysis

Linkage analysis was performed using the Merlin (version 1.1.2) [14] variance components method on MD after adjustment for known covariates. Since variance components linkage analysis is very sensitive to non-normal distributions of traits [18], the MD measurements were transformed to reduce skewness and shrink outliers towards the center of the distributions. A square root transformation was used for PMD, a log transformation for total non-dense area (NDA), and quantile normalization was used for total dense area (DA) since there were several extremely high values for DA. In addition, two extremely large values for NDA were winsorized to the 99^th ^percentile. Trait distributions were unaffected by breast cancer status as mammograms were measured only from the opposite breast, prior to or at a diagnosis. Linear models and generalized additive models were used to model the relationships between the MD scores and known covariates, and the residuals were used in the linkage analysis. Family relationships were not taken into account when fitting the linear models. In general, the expected relationships between MD and covariates were seen. We also estimated heritability in Merlin [14] and in SOLAR version 4.2.7 [19], for the whole sample and for several subgroups. The model used assumed no dominance variance, and allowed for a polygenic effect. There were too few non-Caucasian families to perform variance components linkage analysis separately, however, linkage analysis was repeated in the subgroup of families estimated to be Caucasian by Eigenstrat [15]. To estimate whether our linkage results were exceptional, simulations were performed in Merlin for all autosomes. For these simulations, the phenotypes and pedigree structures were retained, and genotype data were generated by using the estimated allele frequencies. Simulated genotypes were considered unknown if actual genotypes were unknown. Analysis of the simulated data was performed for the residuals from the square root transformation of PMD.

Although this was primarily a linkage study, we calculated evidence for association using the orthogonal test for quantitative traits implemented in QTDT [20] at 5,677 markers. This test focuses on within-family evidence for association, is robust to any differences in allele frequencies between families due to population stratification, and assumes an additive genetic model.

## Results

### Family characteristics

There were 1,616 pedigrees assembled for this study from existing collections, including 4,526 individuals with DNA. Data verification and cleaning included checking for monozygotic twin status (if monozygotic, one twin was removed), gender consistency, whether the mammogram was readable, whether relevant covariate data were available, and whether reported family relationships were consistent with the genotyping. Pedigrees were adjusted when data genotyping supported alternative relationships. A total of 1,415 families were retained for analysis containing 6,638 individuals, of whom 4,993 were women (Additional file 1: supplementary Figure 3).

There were 3,952 genotyped individuals, including siblings, parents and the sisters (3,253) with analysis information (namely genotypes, MD measures, and epidemiologic covariate information). Almost half the families (47%) were recruited from Australia (Additional file 1: supplementary table 2), 35% from Ontario and 18% from California. The families were mostly nuclear, with 77% with three or more women, and 19% with four or more women with complete analysis information. Parental DNA was rarely available; neither of the parents was genotyped in 84% of the families (Additional file 1: supplementary table 2).

### Participant characteristics

Although families were assembled from existing collections that involved a variety of recruitment strategies, the women from the three geographical regions were similar with regard to many of the major characteristics known to impact MD (Table 2). Overall, the average age at mammography was 53 years; 65% of the women were post-menopausal, 38% had a history of hormone therapy use, and 89% had been pregnant at least once. Due to the differences in inclusion criteria of the collections, the percentage of women who had been diagnosed with breast cancer varied across the three sites; with 26% in Ontario, 38% in Northern California and only 5% in Australia. As expected, the percentage of women with breast cancer was higher among those recruited from the Breast Cancer Family Registry sites (Additional file 1: supplementary table 3). In Northern California where recruitment targeted racial/ethnic minority populations [21], the percentage of women self-reporting to be non-Hispanic white was only 44%, whereas it was over 96% in families from Ontario and Australia.

**Table 2:** Description of individuals with complete information

	All sites	Australia	Northern California	Ontario
Women, number	3,253	1,537	579	1,137
Age at mammogram, year	52.8 (8.7)	54.1 (8.9)	51.1 (8.3)	52.0 (8.3)
Self-reported ethnicity, n (%)				
Non-Hispanic white	2,843 (87.4)	1,495 (97.3)	255 (44.0)	1,093 (96.1)
Black	89 (2.7)	0	89 (15.5)	0
East Asian	127 (3.9)	0	119 (20.6)	8 (0.7)
Other/Mixed	189 (5.8)	38 (2.2)	116 (20.0)	35 (3.1)
Age at menarche, year	12.9 (1.5)	13.0 (1.6)	12.8 (1.5)	12.8 (1.5)
Pregnant ever, n (%)	2,889 (88.8)	1,402 (91.2)	496 (85.7)	991 (87.2)
Number of live births, n (%)				
0	489 (15.0)	187 (12.2)	123 (21.2)	179 (15.7)
1	314 (9.7)	115 (7.5)	88 (15.2)	111 (9.8)
2	1,165 (35.8)	534 (34.7)	185 (32.0)	446 (39.2)
3+	1,285 (39.5)	701 (45.6)	183 (31.6)	401 (35.3)
Postmenopausal, n (%)	2,129 (65.5)	1,033 (67.2)	380 (65.6)	716 (63.0)
Past or current hormone therapy use, n (%)	1,248 (38.4)	572 (37.2)	262 (45.3)	414 (36.4)
Body mass index	26.1 (5.4)	26.2 (5.3)	26.4 (6.1)	25.9 (5.1)
Personal history of breast cancer, n (%)	601 (18.5)	84 (5.5)	221 (38.2)	296 (26.0)
PMD	30.3 (17.6)	28.1 (16.1)	33.4 (18.2)	31.6 (18.9)

Risk factors are given as mean (SD) unless otherwise stated. PMD, percent mammographic density.

### Phenotypes

Linear models were fit to the square root of PMD, quantile-normalized DA, and log NDA, adjusting for covariates known to impact MD. The expected relationships with covariates were observed, with MD decreasing with age at mammogram, with each live birth and with menopause, but increasing with hormone therapy use (Table 3; Additional file 1: supplementary table 4). Weight had a non-linear relationship with MD. The same models were also fit for each of the three sites, Australia, Northern California and Ontario, separately, in order to assess whether the relationships between MD and covariates were comparable across sites. Differences in the magnitude of coefficients between sites were, in general, much smaller than the coefficient standard errors (SEs), therefore no obvious differences between sites were apparent (Table 3). Residuals were calculated from these linear models for linkage analysis (PMD_res, DA_res, and NDA_res, respectively).

**Table 3:** Coefficients (standard errors) for covariates in multi-variable linear model predicting the square root of percent mammographic density, overall and by study site

Covariate	All sites together (*n *= 3,253)	Australia (*n *= 1,534)	Northern California (*n *= 578)	Ontario (*n *= 1,166)
Intercept	6.17 (0.72)	5.56 (1.00)	6.62 (1.69)	6.01 (1.33)
Age at mammogram	-0.057 (0.0042)	-0.056 (0.0056)	-0.040 (0.011)	-0.061 (0.0076)
Weight, kg	-0.065 (0.0024)	-0.058 (0.0034)	-0.063 (0.0054)	-0.074 (0.0044)
Height, cm	0.041 (0.0044)	0.041 (0.0060)	0.033 (0.103)	0.047 (0.0081)
Weight squared (centered at 70 kg)	0.00050 (6.9e-5)	0.00049 (1.1e-4)	0.00049 (1.1e-4)	0.00048 (1.4e-4)
Number of live births	-0.103 (0.021)	-0.100 (0.028)	-0.109 (0.047)	-0.096 (0.039)
Postmenopausal status	-0.183 (0.076)	-0.191 (0.109)	-0.056 (0.185)	-0.250 (0.131)
History of hormone therapy use	0.151 (0.0657)	0.133 (0.088)	0.111 (0.172)	0.090 (0.115)

Non-significant covariates (age at menarche, square of mammogram age, square of height) were removed. For the analysis by site, only the covariates in the corresponding overall model were considered.

### Heritability

With the large set of assembled families, it was feasible to evaluate heritability of MD traits for comparison to previous estimates. Heritability estimates (0.50 for PMD, 0.50 for DA, 0.60 for NDA; Table 4) are in general slightly lower than heritability estimated from twin studies [6]. Although heritability estimates for Australia appeared somewhat lower than for the other two sites for residuals based on either the square root of PMD (0.43 for Australia, 0.50 for California, 0.56 for Ontario) or on the DA residuals (Table 4), a 95% confidence interval for the heritability based on all families together includes all of the three site-specific estimates.

**Table 4:** Estimates of heritability from SOLAR (standard error)

	Number of families	PMD_res	DA_res	NDA_res
All families	1,415	0.50 (0.045)	0.50 (0.044)	0.60 (0.043)
Families from Australia	659	0.43 (0.064)	0.43 (0.062)	0.62 (0.063)
Families from Northern California	257	0.50 (0.108)	0.56 (0.105)	0.50 (0.102)
Families from Ontario	499	0.56 (0.075)	0.54 (0.076)	0.60 (0.074)

PMD_res, DA_res and NDA_res are residuals from linear models adjusting the square root of PMD, quantile-normalized dense area, and log non-dense area, respectively, for covariates known to affect MD. MD, mammographic density; PMD percent mammographic density.

### Genetic marker characteristics

After removing inconsistent and failed markers, 5,677 SNPs remained for analysis (See Materials and Methods). Tests for Hardy-Weinberg equilibrium (HWE) [22] were performed using one randomly chosen genotyped woman per family. When only Caucasian families are included in these tests (excluding 105 families containing 315 individuals), there is no evidence for deviation from HWE (Additional file 1: supplementary figure 5). For this subset analysis, Caucasian ethnicity was defined as individuals who clustered with the HapMap CEU individuals in Eigenstrat PC analysis [15].

### Genome-wide Linkage

#### Primary analysis

**Figure 1:**
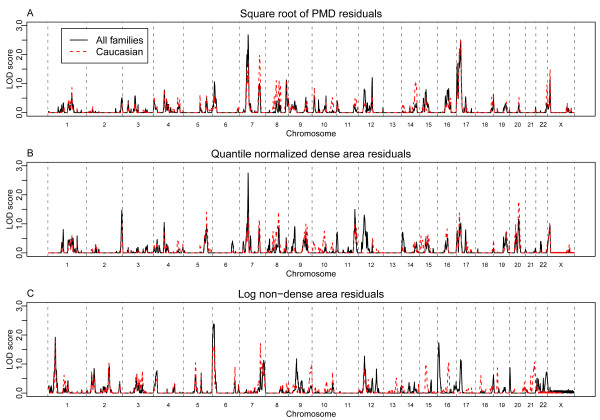
**LOD score linkage tests across all chromosomes, for all families and Caucasian families**. LOD scores at SNPs in the linkage panel are plotted against their genetic distance (in cM) along each chromosome; chromosome boundaries are marked by vertical dashed lines. Results are shown for (**A, B **and **C**) all families (solid black traces) and also for Caucasians (dashed red traces) for phenotypes indicated. SNP, single-nucleotide polymorphism.

Logarithm of odds (LOD) score linkage plots for PMD, DA and NDA are shown in Figure 1 (panels A, B and C, respectively) for the autosomes and the X chromosome. The maximum LOD score from the genome-wide scan for PMD was 2.69 on chromosome 7p14.1-p13 (at 63.5 cM; 46.5 Mb, NCBI Build 36.3) with a 1-LOD drop interval from 58.5 cM to 67.2 cM. A peak at the same location was also observed for DA (peak LOD 2.69 at 64.9 cM, 1-LOD interval 60.0-68.6). Another peak (LOD = 2.44) was also found on chromosome 17 for PMD. Among 100 autosome-wide simulations under the null hypothesis, more than half of the simulations indicated a maximum LOD score larger than 2.5 and one third had a LOD score over 3.0 with the empirical 5% significance level being 4.4. Therefore, linkage peaks of the magnitude that we found can be expected under random inheritance patterns, and it is unclear if these peaks reflect true genetic linkage signals. However, our results do provide evidence for suggestive linkage, since our simulations showed that the empirical threshold for suggestive linkage in these data would be 2.4.

#### Additional analyses

We then performed several different analyses of ancillary phenotypes and subgroups to investigate whether the evidence for linkage was stronger under different assumptions.

##### Weight and height

Weight and height are partially determined by genetic factors. We considered the possibility that by including weight and height in the models for calculating residuals, we may have minimized genetic effects due to pleiotropy. Linkage was re-estimated using residuals to models containing all predictors except weight and height; no additional linkage peaks were identified. Conversely, we also considered the possibility that we were not adequately capturing the relationships between PMD and weight, height, and age at mammogram in our linear models. We therefore fitted generalized additive models [23] for these variables, including a bivariate smooth for weight and height, but again no additional linkage peaks were identified.

##### Age

**Figure 2:**
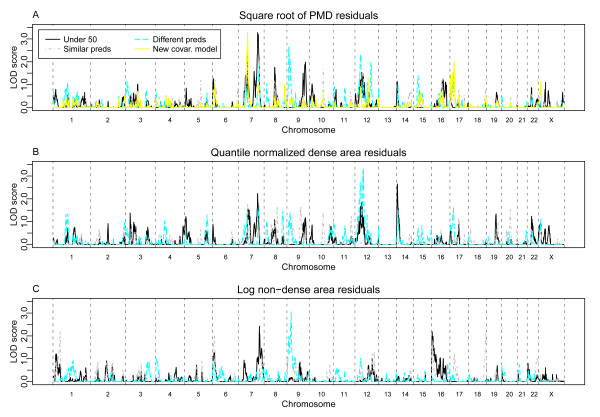
**Linkage re-analysis with an alternate covariate model for PMD or various subgroups of families**. Linkage results are shown for (**A**) all families with an alternate covariate (new covar.) model for PMD (yellow trace) and (**A, B **and **C**) for families containing at least two sisters diagnosed before age 50 (black traces), families where sisters have very similar predictions (preds) of MD (grey traces), or families where sisters have very different predictions of MD (turquoise traces) for phenotypes indicated. MD, mammographic density; PMD, percent mammographic density.

It has been often argued that the influence of genes, relative to the environment, will be larger for early onset features or diseases. Further, it has been noted in at least one study that three SNPs that had been reported to be associated with breast cancer were marginally associated with MD, but only when women with younger ages (pre-menopausal) were considered [24]. We examined the evidence for linkage in families with at least two sisters who were under 50 years at the time of their mammogram, see Figure 2, to see whether linkage signals appear stronger in this subgroup. In analysis including only this subset of 513 pedigrees meeting this age criterion, a modest peak seen for the PMD phenotype on the q arm of chromosome 7 increased in strength (compare Figure 1A to Figure 2A), with a peak LOD score of 3.2 (140.7 cM, 1-LOD drop interval 137.7 - 147.3, 7q32.3-7q34). It is notable that this increased signal was identified among a much smaller number of families (empirical genome-wide significance level *P *= 0.12) highlighting this chromosomal region.

##### Menstrual, reproductive and hormonal variables

MD decreases with age and is influenced by many factors that alter endogenous sex hormone levels, such as the number of pregnancies, hormone therapy use, and menopausal status. Although in our primary analysis, we had analyzed residuals to linear models adjusted for these factors, we hypothesized that the magnitude of the potential effect of a locus on MD measurements could vary as a function of these reproductive and hormonal variables. To explore this hypothesis, we used our linear models with covariates to obtain a predicted PMD value for each individual, and then we calculated the smallest difference in predicted PMD among all pairs of sisters in each family. We then divided the families into two equally-sized groups based on this predicted PMD difference. Hence, in one group, age, menstrual and reproductive values led to predictions of more similar PMD for the sisters, whereas in the other group, larger PMD differences were predicted by the covariate model; this concept is illustrated in Additional file 1: supplementary figure 6. Evidence for linkage was calculated in these two subgroups, and results are also shown in Figure 2. In families containing sisters with dissimilar predictions, we saw a linkage peak for the DA phenotype on chromosome 12p11.22-q13.11 (max LOD = 3.30 between *rs2061192 *and *rs10785424*, 1-LOD drop interval from 56.0 cM - 64.0 cM, Figure 2B). Linkage in this subset implies that sharing of markers in this region is associated with more similarity in DA, among families where MD measures are expected to be quite variable due to covariates. Although this analysis used the total dense area phenotype rather than PMD, a reduced signal was also seen when we analyzed PMD (Figure 2A). DA, like PMD, is positively associated with breast cancer risk and may have genetic determinants that are not shared with the non-dense area of the breast. Our signal is within a broad linkage signal spanning an approximately 90 cM region (2-LOD drop interval) described by Vachon and colleagues [25]. Their signal appeared to contain two peaks, one with a maximum LOD score of 2.47 with a 1-LOD drop interval from 19 cM to 46 cM and a second peak with a maximum LOD score of 2.45 with a 1-LOD drop interval from 55 cM to 101 cM (Vachon, personal communication). Vachon and colleagues employed a model with PMD measures adjusted for covariates but did not evaluate dense area as a separate phenotype.

##### Refining the PMD phenotype

To explore the impact of phenotype-covariate modelling on the linkage results, we refit our linear models for the square root of PMD phenotype using a different parameterization that was suggested by the Pike model [26]. In light of this model, which relates cumulative exposure to 'breast tissue age' with breast cancer incidence rates, breast tissue age can be expected to be greatest at menarche, to decrease with each pregnancy, and to decline rapidly through the menopausal period. Therefore, we defined separate covariates to estimate the decrease in PMD with each year at each stage in life (that is, (1) between menarche and the first live birth, (2) during the child-bearing years, (3) post child-bearing years, and (4) post-menopause). Covariates for the number of pregnancies and use of hormonal therapy were included. In addition, we fit these models using generalized estimating equations to adjust for the effects of within-family correlation when estimating parameters. The signal on chromosome 7p is enhanced by using these residuals for the phenotype, with the peak at *rs1029482 *rising to 3.29 (1-LOD interval 59.2 - 67.3 cM) (Figure 2A, new covariate model). Eighteen percent of our simulations had a peak LOD score over 3.5, thus the evidence for linkage to this locus is still not genome-wide 'significant' [27].

#### Association analysis

**Figure 3:**
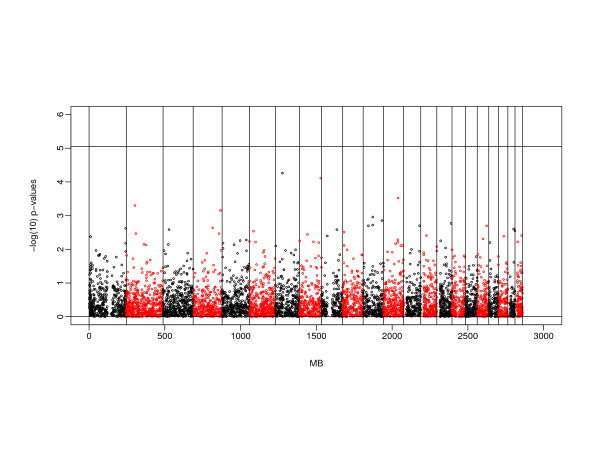
**Manhattan plot of association test results for the square root of PMD residuals**. *P*-values (-log10) of association tests using the orthogonal model of QTDT are plotted against physical position across the chromosomes. A Bonferroni correction for 5677 tests would lead to a threshold of 5.05 for significance at α = 0.05. PMD, percent mammographic density.

The smallest *P*-values obtained from the tests of within-family association [20] are shown in Table 5; all results for the square root of PMD residual phenotype are shown in a Manhattan plot (Figure 3; see also Additional file 1: supplementary figure 7 for a QQ plot). Using a simple Bonferroni correction for 5,677 markers, tests for association would be considered significant at 8.8 × 10^-6^. Although none of our markers showed significance at this threshold, the most significant result (*rs723149*; 67.7 cM; *P*-values 5.5 x10^-5 ^and 2 × 10^-4 ^for PMD and DA, respectively) occurred on chromosome 7 near our linkage peak. To pursue this further, sliding two- and three-marker haplotypes were tested for association using FBAT [28] around this marker, and a two marker haplotype consisting of allele A at *rs1486155 *and allele A at *rs723149 *showed a stronger association at *P *= 6.9 × 10^-7^. There was weak linkage disequilibrium between these two markers (D' = 0.20; 281 Kb). The minor allele frequency at *rs723149 *varies across populations http://hapmap.ncbi.nlm.nih.gov, however our test of association using QTDT examines within-family patterns of allele transmission and is robust to population stratification. Furthermore, similar results were obtained when we analyzed the subset of Caucasian families (not shown).

**Table 5:** Selected tests of within-family association with at least one phenotype with *P*-value ≤0

Chromosome	Marker	Nucleotide position	Minor allele/MAF in our data	PMD_res	DA_res	NDA_res	Allele with higher MD
**1**	rs1526480	91,209,986	0.39 (G)	0.041	0.71	**4.00e-4**	A
**2**	rs715271	57,133,410	0.32 (C)	**5.00e-4**	0.0019	0.024	A
**7**	rs723149	46,543,581	0.43 (A)	**5.50e-5**	**2.00e-4**	0.035	A
**8**	rs4317547	141,285,098	0.27 (A)	**7.77e-5**	.0016	**2.00e-4**	A
**12**	rs1012315	97,639,144	0.29 (A)	**3.00e-4**	7.00e-4	0.018	G
**14**	rs764602	49,480,419	0.48 (A)	0.022	0.35	**3.00e-4**	G
**14**	rs1959064	57,044,005	0.32 (G)	0.036	**2.00e-4**	0.045	A
**20**	rs7272911	35,946,239	0.30 (A)	0.0041	0.091	**1.00e-4**	G

In order to see whether the linkage signal on chromosome 7p was explained by this association, we imputed haplotypes near *rs723149 *by using PLINK [13], and created a covariate based on the probable count of haplotype AA at *rs1486155 *and *rs723149*. This covariate was then included in the linkage analysis, but was found to have minimal impact. The LOD score fell by 0.21 for PMD_res, from 2.69 to 2.48.

Genotype information at *rs723149 *of additional sets of women with MD measurements and covariate information was available. These included a non-overlapping set of 235 women from Ontario and Australia [29] selected for extreme values for MD that had been genotyped with the Illumina Sentrix Human 1M BeadChip platform and an additional group of 789 unrelated women from Ontario (with characteristics as for the recruited family participants) genotyped with a Taqman^® ^SNP Genotyping Assay specifically for rs723149. No evidence for association with PMD was observed in these 1,024 women (MAF = 0.42, linear regression with 0,1, 2 coding for number of copies of allele A, β = -0.077 (SE = 0.083), *P *= 0.35, for association with the square root of PMD adjusted for age, age squared, weight, weight squared, height, height squared, parity, and menopausal status).

## Discussion

Phenotype definition is critical in linkage analysis. The extent of MD can be measured by the percent of the breast that is dense or by the total area that is dense. DA is highly correlated with PMD, but has a skewed distribution. Nevertheless, after transformation and adjustment for covariates, repeated reads of the same mammogram showed very high correlations for DA and for PMD. Therefore, we examined the evidence for linkage to both these phenotypes, and found evidence for suggestive linkage at multiple locations. Furthermore, since MD is known to vary through life, genes that appear to influence MD in mid-life could reflect early influences on MD at the time the breast forms in adolescence, or through subsequent changes or rates of change with increasing age, parity and the menopause, or through influences on both formation and age change in MD [30]. Our study was designed so that there was rarely more than five years difference between the ages at mammogram for sister pairs in a family; hence the study was partially matched within families for the large effect of age on MD. In addition, MD measures were adjusted for age prior to linkage analysis.

Given the number of study participants and available pedigree information, we had estimated that we would have 80% power to obtain a LOD score in the range of 3 with a locus that could explain 25% of the heritability of PMD. Despite the large collection of families and careful consideration of mammographic density parameters, our primary linkage analysis did not yield LOD scores that exceeded desired thresholds set by genome-wide gene-dropping simulations. With different modeling conditions, three loci on chromosomes 7p14.1-p13, 12p11.22-q13.11, and 7q32.3-q34, showed LOD scores that approach or exceed 3. However, given that these results were obtained after multiple analyses, these signals should be considered as 'suggestive'.

**Chromosome 7p: **With the development of a model for covariate effects that was inspired by Pike and colleagues [26], evidence for linkage increased on chromosome 7p, with a maximum LOD score of 3.29. This increase associated with a more careful phenotype definition is suggestive that there may be loci influencing aspects of MD or MD changes, but also that the ideal parameterization for MD is still unknown. The 1-LOD drop interval bounded by *rs1949880 *and *rs2054789 *corresponds to a 7.2 Mb region containing 72 genes, including small clusters of snoRNA and piRNA genes toward its proximal boundary. Of interest at the proximal boundary, are the insulin-like growth factor binding protein genes, *IGFBP1 *and *IGFBP3*, both of which have been hypothesized to be involved in mammographic density (and in breast cancer and other cancers) and have been considered in previous association studies examining MD phenotypes [31-35]. Of these, two studies included more than 1,000 unrelated women [32,3] with investigation of the common genetic variation in these genes, but they did not reveal consistent evidence of association with MD phenotypes. Also, a recent meta-analysis of 4,877 women did not identify association with MD in this region [29]. As linkage analysis may be less sensitive to allele frequency issues, these genes remain as interesting candidates. Another gene of potential interest within this region includes the ras-related gene, v-ral simian leukemia viral oncogene homolog A (*RALA*) with its implicated role in signalling and growth.

**Chromosome 7q: **When we examined families containing younger sisters (two sisters with mammograms under age 50 years), the peak on chromosome 7q became stronger. This peak with a 1-LOD drop interval bounded by *rs4728251 *and *rs1476640*, corresponds to 9.6 Mb containing 69 genes. Phenotypes at younger ages are expected to display a stronger genetic component [36] but there was no evidence that PMD h^2 ^varied by age in our previous twin studies [30,37], and thus age should continue to be considered in future studies. A gene of potential interest within this region is a member of the *RAS *oncogene family, *RAB19*.

**Chromosome 12: **On chromosome 12, a maximum LOD score of 3.3 was seen in families where the sisters would be expected to have dissimilar DA, after adjustment for factors affecting sex hormone levels. Such predicted differences could be due to: differences in the numbers of pregnancies; ages at menopause or menarche; weight; or height. Linkage in this context would identify families where phenotypes are more similar than expected, and this could imply a larger potential genetic effect. The 1-LOD interval of our linkage signal, bounded by *rs1909160 *and *rs1978161 *corresponds to a large physical distance of 16.7 Mb encompassing the centromere and contains 80 genes. Genes of potential interest within this region include the vitamin D receptor (*VDR*) and collagen type II (*COL2A1*) given their association to breast biology.

We did not detect any significant evidence for linkage on chromosome 5 as was reported in a previous study of 89 multi-generation pedigrees [25], which, like our study, included mostly Caucasian women; however, there may have been differences in the family ancestries or characteristics of the source populations that are not obvious.

That high mammographic density is associated with risk for breast cancer motivated our study, with anticipation that some gene determinants of MD may be candidates for involvement or development of malignancy [29]. Many groups have pursued genome-wide linkage studies for breast cancer using non-*BRCA1/2 *high risk families, more recently with families of confined ancestries (see [38], and within) after family sets of diverse origins did not yield strong linkage signals [39,40]. Suggestive evidence for a chromosome 7 locus was identified in one study [39], however, this region is more proximal occurring at 7q21.11-q21.3, and does not overlap with the suggestive MD locus detected in our younger women set. Large scale association studies have also been carried out, several with discovery or first-stage study sizes that exceed 1,000 cases of breast cancer [41-46] leading to loci for consideration, with considerable family risk remaining to be explained [45]. We note that none of our suggestive linkage peaks observed coincide with the candidate genes and their local SNPs that reached genome-wide significance in these large studies.

## Conclusions

Despite a reliable and plausible intermediate phenotype for breast cancer risk, we were unable to identify new loci with a strong influence on MD, nor could we confirm a previously reported region on chromosome 5p13-p14. However, we did identify signals on chromosomes 7p, 7q, and 12 that warrant further investigation. Notably, the evidence for linkage among younger women on 7q, as well as thepeak on chromosome 12 overlapping a previously-identified region may prove to be interesting.

## Abbreviations

CIDR: Center for Inherited Diseases Research; DA: dense area; DZ: dizygous; GWAS: Genome-Wide Association Study; HWE: Hardy-Weinberg Equilibrium; LD: linkage disequilibrium; LOD: logarithm of odds; MAF: minor allele frequency; MD: mammographic density; MZ: monozygous; NDA: non-dense area; PC: principal components; PCA: principal components analysis; PD: percent density; PMD: percent mammographic density; SE: standard error; SNP: single-nucleotide polymorphism.

## Competing interests

The authors declare that they have no competing interests.

## Authors' contributions

LL, EMJ and CA coordinated the collection of mammograms in Ontario, Northern California and Australia, respectively. LL and CA digitized the mammograms and NFB read all mammogram images. LL, CA, VK and AS coordinated and organized the epidemiological information. MCS, EMJ, AS, RVP, ES and JMR coordinated, collected, and organized the DNA samples. CMTG carried out the linkage analysis, heritability analysis and drafted the manuscript. CMTG and AD performed the association analysis. NFB, ADP, ILA, LJM, MCS, EMJ, JLH and JMR conceived and designed the study. All authors read and approved the final manuscript.

## Authors' information

CMTG is the Pharmaprix Weekend to End Cancer Career Scientist. ADP holds a Canadian Research Chair in the Genetics of Complex Diseases. LJM is a recipient of a New Investigator Award from the Canadian Institutes of Health Research. MCS is a National Health and Medical Research Council (NHMRC) Senior Research Fellow and a Victorian Breast Cancer Research Consortium Group Leader. JLH is an Australian Fellow of the NHMRC and a Victorian Breast Cancer Research Consortium Group Leader.

## Supplementary Material

Additional file 1 **Supplementary Figures and Tables**. Supplementary Table 1. Recruitment of families for linkage with details of Ontario studies. Supplementary Table 2. Family characteristics. Supplementary Table 3. Breast cancer rates among women with complete data, by study type. Supplementary Table 4. Coefficients (standard errors) for covariates in multi-variable linear models predicting DA and NDA, all three sites together. Supplementary Figure 1. Participant recruitment by site. Supplementary Figure 2. Reliability of mammographic density scoring. Supplementary Figure 3. Data cleaning for genetic analysis. Supplementary Figure 4. Principal component analysis of study participants. Supplementary Figure 5. QQ plot of tests of Hardy-Weinberg equilibrium. Supplementary Figure 6. Illustration of the division of families into two groups as a function of predicted mammographic density. Supplementary Figure 7. QQ plot of association tests for the residuals from a linear model for the square root of PMD. Click here for file
