# Supplementary material for: A genome-wide linkage study of mammographic density, a risk factor for breast cancer
Source: Breast Cancer Res. 2011 Dec 21;13(6):R132. doi: 10.1186/bcr3078 (PMC3326574; doi:10.1186/bcr3078)

A genome-wide linkage study of mammographic density, a risk factor for breast cancer

Supplementary Materials: Figures and Tables

Supplementary Table 1. Recruitment of families for linkage with details of Ontario studies.

Supplementary Table 2. Family characteristics.

Supplementary Table 3. Breast cancer rates among women with complete data, by study type.

Supplementary Table 4. Coefficients (standard errors) for covariates in multi-variable linear models predicting DA and NDA, all three sites together.

Supplementary Figure 1. Participant recruitment by site.

Supplementary Figure 2. Reliability of mammographic density scoring.

Supplementary Figure 3. Data cleaning for genetic analysis.

Supplementary Figure 4. Principal component analysis of study participants.

Supplementary Figure 5. QQ plot of tests of Hardy-Weinberg equilibrium.

Supplementary Figure 6. Illustration of the division of families into two groups as a function of predicted mammographic density.

Supplementary Figure 7. QQ plot of association tests for the residuals from a linear model for the square root of PMD.

Supplementary Table 1. Recruitment of families for linkage with detail of Ontario studies.

Study Name	No. of families (%)	No. of women with complete data for analysis (%)
**All Families together**	**1415 (100)**	**3253 (100)**
Australian site of the BCFR	70 (5.0)	174 (5.4)
Australian Twin & Sister Study	589 (41.6)	1363 (41.9)
Northern California site of the BCFR	257 (18.2)	579 (17.8)
Ontario site of the BCFR	246 (17.4	561 (17.3)
Ontario Breast Screening Program, Twins	47 (3.3)	95 (2.9)
Twin Research Program (Ontario)	84 (5.9)	193 (5.9)
Weekend to End Breast Cancer (Ontario)	103 (7.3)	249 (7.7)
Young Women's study (Ontario)	19 (1.3)	39 (1.2)

Supplementary Table 2. Characteristics of the families.

		All families	Australia	California	Ontario
	**No. in family**				
**Number of families**		**1415**	**659**	**257**	**499**
Number of women in family with complete data[1]	2	331	93	24	214
	3	811	421	190	200
	4	198	109	34	55
	5	57	26	7	24
	>=6	18	10	2	6
Number of genotyped parents	0	1191	638	157	396
	1	164	15	66	83
	2	60	6	34	20

[1] Complete data refers to a) genotyping, b) MD measurement, and c) relevant covariate data

Supplementary Table 3. Personal history of breast cancer among women with complete data, by study type.

Site	Women from the BCFR sites		Women from other studies	
	No. of women	With breast cancer (%)	No. of women	With breast cancer (%)
**Australia**	174	11 (6.3)	1363	73 (5.4)
**Northern California**	579	221 (38.2)	0	--
**Ontario**	561	180 (32.1)	576	116 (20.1)
Ontario Breast Screening Program, Twins			95	1 (1.1)
Twin Research Program (Ontario)			193	81 (42.0)
Weekend to End Breast Cancer (Ontario)			249	33 (13.3)
Young Women's Study (Ontario)			39	1 (2.6)

Supplementary Table 4. Coefficients (standard errors) for covariates in multi-variable linear models predicting DA and NDA, all three sites together.

Covariate	Model for DA	Model for NDA
**Intercept**	0.95 (0.45)	11.86 (0.20)
**Age at mammogram**	-0.024 (0.025)	0.014 (0.0011)
**Weight, kg**	-0.0037 (0.0012)	0.033 (6.5e-4)
**Height, cm**	0.0061 (0.0027)	-0.0187 (0.0012)
**Weight squared (centered at 70kg)**	(Not in model[1])	-0.00028 (1.9e-5)
**Number of live births**	-0.047 (0.012)	0.021 (0.0055)
**Postmenopausal status**	-0.082 (0.046)	0.031 (0.021)
**History of hormone therapy use**	0.070 (0.039)	-0.038 (0.017)
**Age at menarche**	-0.022 (0.011)	-0.011 (0.0049)

[1] Weight squared was not significantly associated with DA and was removed from the final model

Supplementary Figure 1. Participant recruitment by site.

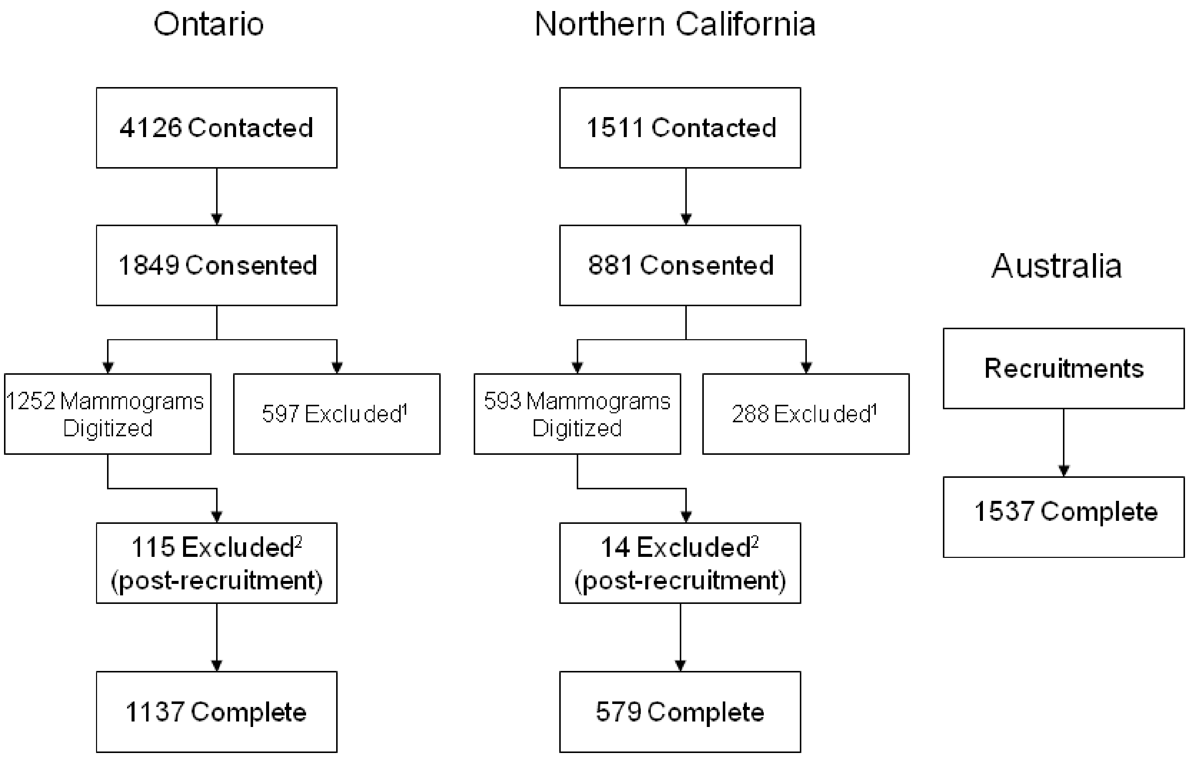

Recruitment of women and mammograms were carried out with the aim of obtaining sister sets with mammograms taken such that their ages were within five years. Approaches in Ontario involved contacting women and obtaining consent for obtaining mammograms as they were already participating ongoing collections (BCFR from Ontario Cancer Genetics Network, Ontario Breast Screening Program, Twin Research Program, Weekend to End Breast Cancer) or were recruited specifically for genetic studies in mammography density with blood collection (Young Women's Study). Participants in the BCFR from Northern California were contacted to obtain consent for mammograms, and DNAs were obtained from Coriell Institute for Medical Research. In Australia, families were selected for the linkage study with consent as DNA and mammograms were available from a minimum of two sisters per family that were recruited in the Australian Mammographic Twins & Sisters Study or the Australian site of the BCFR [9].

[1]Excluded during mammogram collection (Ont/Cal): no pre-diagnostic mammogram (155/112), breast augmentation/reduction(39/32), inability to match mammograms on age (71/83), lost or destroyed films (34/36).

[2]Excluded post-recruitment due to biospecimen (DNA) collection issues.

Supplementary Figure 2. Reliability of mammographic density scoring.

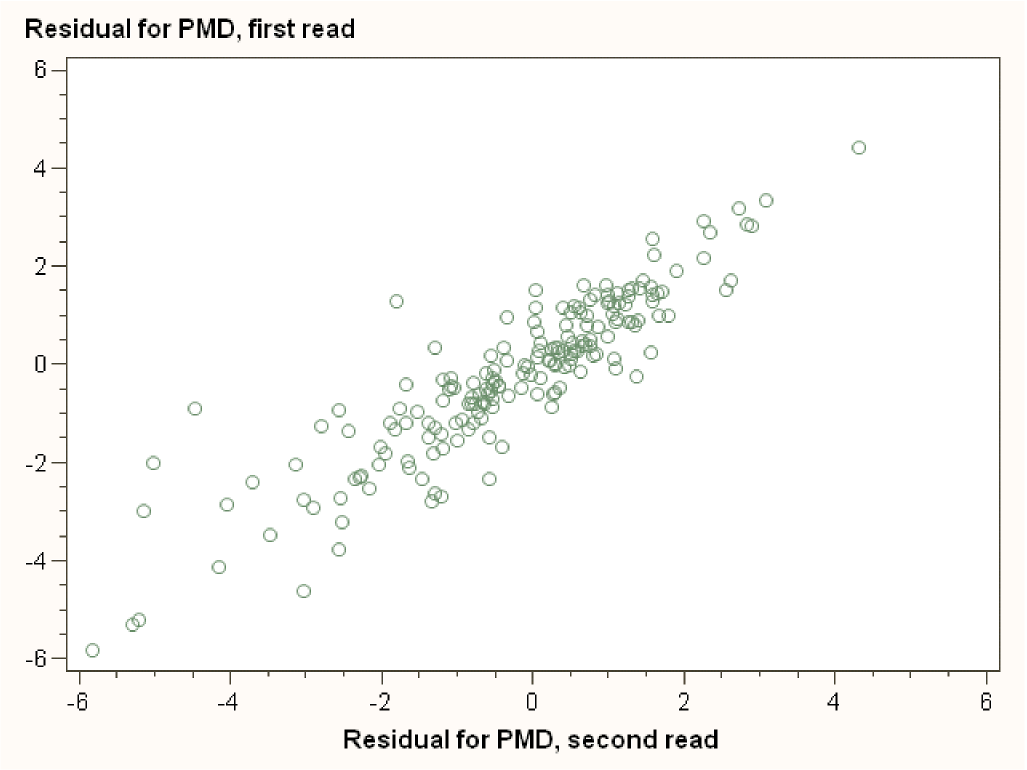

The PMD for the first read is plotted against the PMD from the second read, for images randomly selected to be read twice, as described in the Materials and methods.

Supplementary Figure 3. Data cleaning for genetics analysis.

1616 families, including 4526 individuals with DNA, assembled from Ontario, Northern California and Australia

↓

219 individuals excluded during data collection and assembly:

- 7 duplicates
- 4 poor quality DNA
- 2 mammograms were from another woman
- 2 DNA samples with inconsistent gender
- 1 father's DNA was inconsistent with his daughters
- 113 individuals who had a MZ twin and, in addition to another sister
- 42 pairs of MZ twins with no additional sisters, with 4 mothers and 2 brothers (90 individuals)

87 families were excluded

↓

111 families excluded due to incomplete data for mammograms or covariates

↓

After genotyping: 56 families required adjustment to pedigree structures

- Half siblings, not full siblings (46 families)
- Apparently unrelated children (3 families)
- Nuclear families that were found to be cousins (5 families)
- One sample mix-up (involving 2 families)
- Half-sib pair appeared to be a full sib pair (1 family)

3 families excluded (not informative for linkage)

↓

1415 families remained for analysis
3952 individuals with genotypes
6638 individuals in total (including those connecting pedigrees)
3253 women with genotypes, mammograms, and covariate data

The cleaning of samples and families prior to the application of linkage analysis was carried out as described in the flowchart.

Supplementary Figure 4. Principal component analysis of study participants.

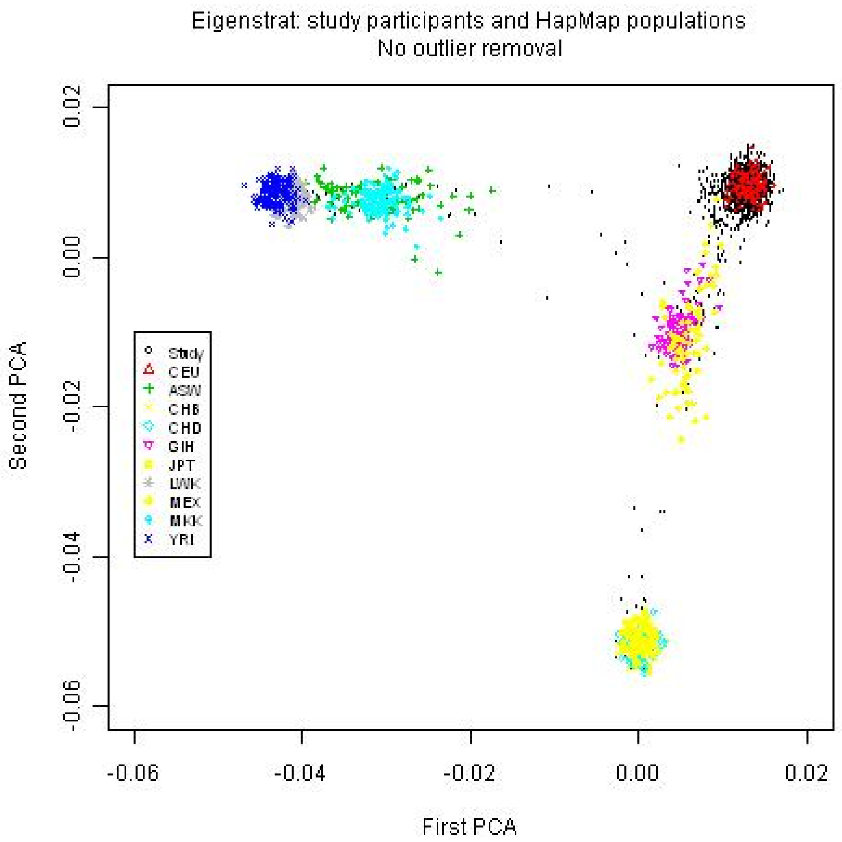

Population structure was estimated with one individual per family with HapMap samples [15]. First principal component is plotted against the second principal component. The majority of study participants cluster with the CEU HapMap samples.

Supplementary Figure 5. QQ-plot of tests of Hardy-Weinberg equilibrium.

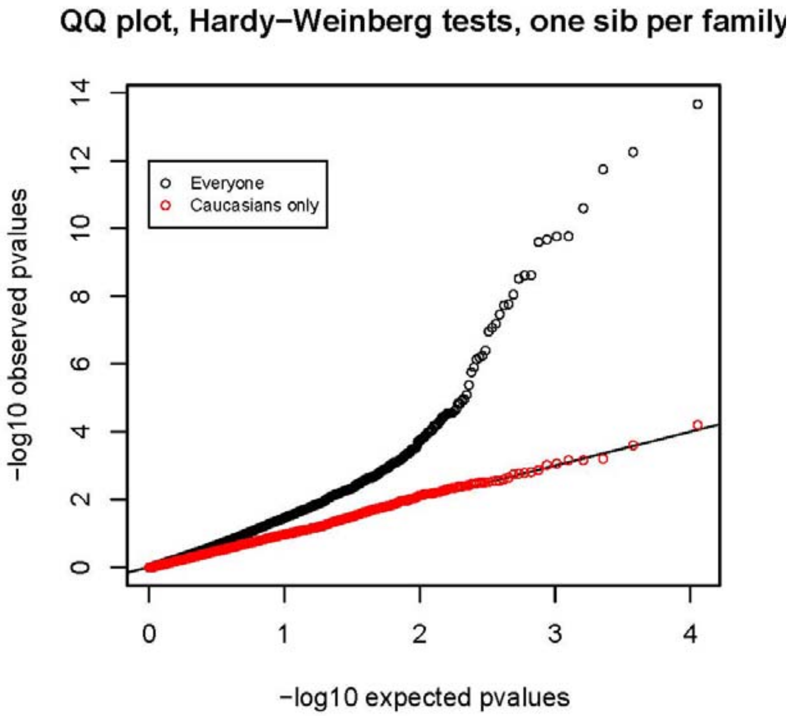

Tests of Hardy-Weinberg equilibrium were based on one randomly chosen woman per family. Caucasian families were inferred, based on clustering with HapMap CEU individuals in Eigenstrat PC analysis [15].

Supplementary Figure 6. Illustration of the division of families into two groups as a function of predicted mammographic density.

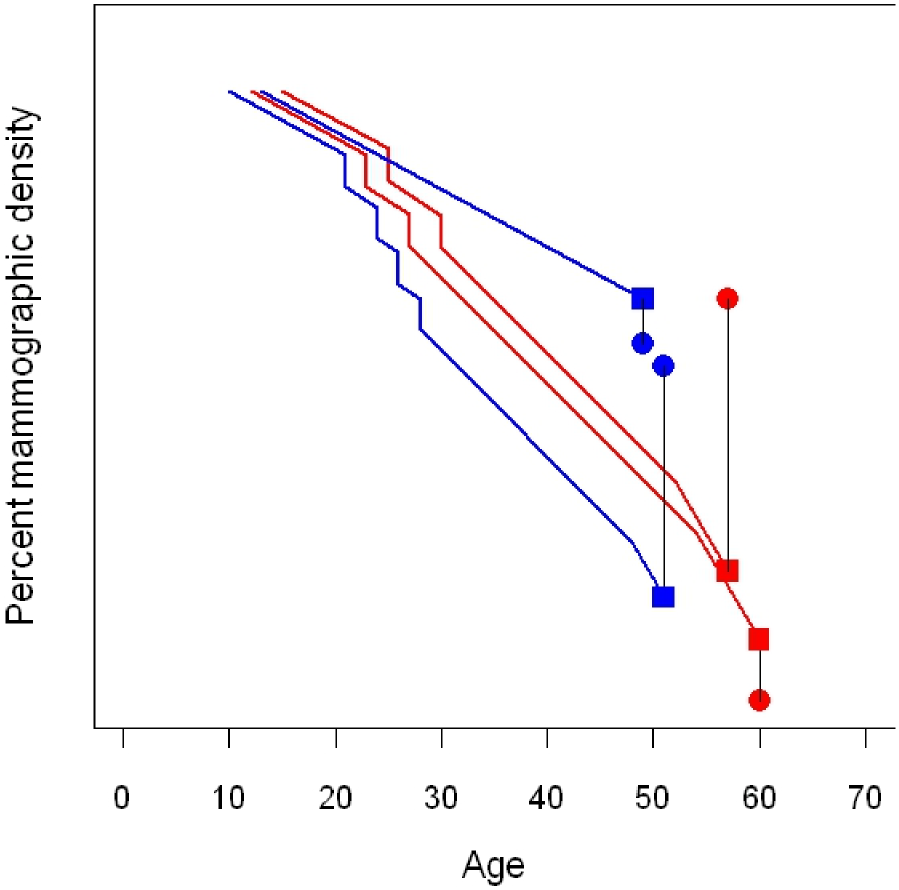

Hypothetical changes in PMD as a function of age, pregnancies and menopause are illustrated for two sets of sister pairs. Each line shows a potential trajectory of PMD changes throughout life, based on the coefficient estimates from our alternative covariate model. The stepped decreases in lines, demonstrate the assumed effect of pregnancies on PMD. The steeper slopes beyond approximately age 50 represent the anticipated rapid decrease in PMD after menopause. Sisters are represented by the same colour, squares are predicted PMD, and circles are observed PMD values. Predictions depend on the age at menarche, number of children, mother's age at the beginning and end of child-bearing years, and age at menopause. The family with sisters with red symbols would be assigned to the group with similar predictions since the two red squares have similar PMD values, but different PMD values were observed for these two women. In contrast, the sisters of the family with blue symbols would be assigned to the group with different predictions.

Supplementary Figure 7. QQ plot of association tests for the residuals from a linear model for the square root of PMD.

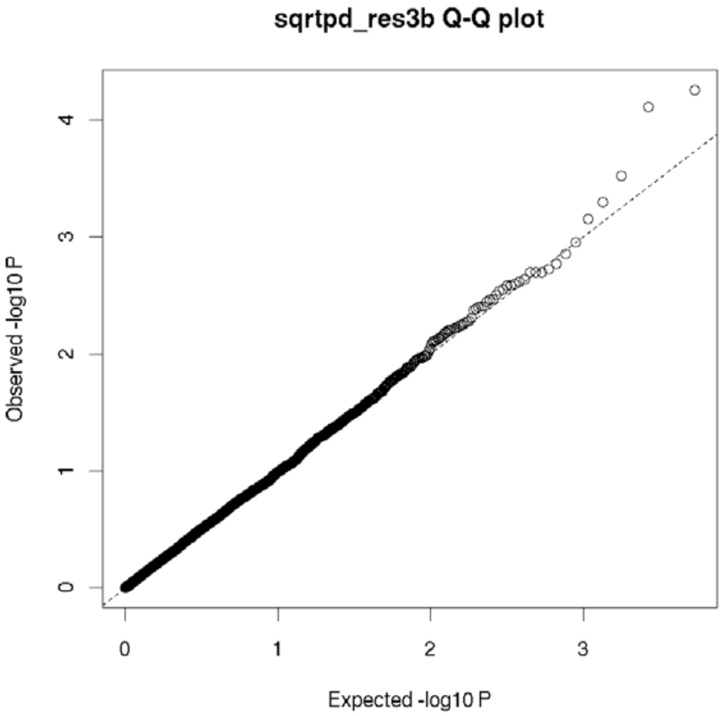

Supplement: Additional file 1 — Supplementary Figures and Tables. Supplementary Table 1. Recruitment of families for linkage with details of Ontario studies. Supplementary Table 2. Family characteristics. Supplementary Table 3. Breast cancer rates among women with complete data, by study type. Supplementary Table 4. Coefficients (standard errors) for covariates in multi-variable linear models predicting DA and NDA, all three sites together. Supplementary Figure 1. Participant recruitment by site. Supplementary Figure 2. Reliability of mammographic density scoring. Supplementary Figure 3. Data cleaning for genetic analysis. Supplementary Figure 4. Principal component analysis of study participants. Supplementary Figure 5. QQ plot of tests of Hardy-Weinberg equilibrium. Supplementary Figure 6. Illustration of the division of families into two groups as a function of predicted mammographic density. Supplementary Figure 7. QQ plot of association tests for the residuals from a linear model for the square root of PMD. [file bcr3078-S1.PDF]
